# Melatonin exhibits partial protective effects against gemcitabine- and cisplatin-induced kidney and reproductive injuries in mice

**DOI:** 10.18632/aging.205307

**Published:** 2023-12-14

**Authors:** Shao-Chuan Wang, Hsuan-Chih Hsu, Ya-Chuan Chang, Chia-Ying Yu, Chien-Te Liu, Wen-Wei Sung

**Affiliations:** 1 Department of Urology, Chung Shan Medical University Hospital, Taichung 40201, Taiwan; 2 School of Medicine, Chung Shan Medical University, Taichung 40201, Taiwan; 3 Institute of Medicine, Chung Shan Medical University, Taichung 40201, Taiwan

**Keywords:** melatonin, hormone, testosterone, sperm, chemotherapy

## Abstract

Cisplatin has the potential to cause kidney and reproductive organ injuries, prompting the search for protective agents against cisplatin-induced toxicity. Melatonin, an antioxidant hormone, has shown promise in mitigating oxidative stress in various organs. However, its protective effects on cisplatin-induced kidney and reproductive injuries have not been extensively investigated. The aim of this study was to explore the potential protective effects of melatonin on cisplatin-induced kidney and reproductive injuries when administered in combination with gemcitabine in mice. Male C57BL/6 mice were subjected to a seven-week treatment with gemcitabine plus cisplatin, with or without melatonin intervention. The testis, epididymis, and kidney were assessed through histological analysis and measurement of blood parameters. Treatment with cisplatin led to a significant reduction in testicular weight, histological abnormalities, and alterations in reproductive hormone levels. Melatonin exhibited a slight protective effect on the testis, with higher doses of melatonin yielding better outcomes. However, melatonin did not reverse the effects of cisplatin on the epididymis. Administration of melatonin before and during treatment with cisplatin plus gemcitabine in mice demonstrated a modest protective effect on testicular injuries, while showing limited effects on epididymal injuries. Serum creatinine levels in the group treated with gemcitabine plus cisplatin treatment and high-dose melatonin approached those of the control group, indicating a protective effect on the kidney. These findings underscore the potential of melatonin as a protective agent against cisplatin-induced kidney and reproductive injuries and emphasize the need for further research to optimize its dosage and evaluate its long-term effects.

## INTRODUCTION

Cisplatin is a widely used anticancer drug in the treatment of various types of carcinomas and sarcomas [1, 2]. It contains a platinum base at its core and exerts its therapeutic effects by entering the cell and forming crosslink complexes on DNA, thereby increasing the accessibility of DNA to solvents [3]. These platinum-DNA adducts initiate the apoptotic pathway, leading to cell death [4]. Furthermore, the cis-diamminedichloro platinum center of cisplatin induces oxidative stress by promoting the generation of reactive oxidative species (ROS). The level of ROS is positively correlated with both the concentration and duration of exposure to cisplatin [5]. While tumors have a high capacity to tolerate ROS, the accumulation of ROS within tumor cells can eventually trigger intrinsic and extrinsic apoptosis, as well as ferroptosis [6]. Cisplatin was initially approved for clinical use in testicular cancer by the United States Food and Drug Administration in 1978. Subsequently, it has also been approved for the treatment of non-small cell lung cancer, ovarian cancer, breast cancer, and head and neck cancer [7–11].

Although cisplatin has a wide range of uses in cancer treatment, its toxicity in various organs, including the liver, heart, kidneys, hearing system, and peripheral nerves, limits its therapeutic potential [1, 2, 12]. Clinically, 25–40% of patients undergoing cisplatin treatment show nephrotoxicity characterized by a decreased glomerular filtration rate, elevated serum creatinine levels, and hypomagnesemia [13–15]. Cisplatin-induced acute kidney injury (AKI) is associated with the following pathophysiological processes: proximal tubular injury, oxidative stress, inflammation, and vascular injury [16]. Under cisplatin stress, p53 is activated and directly or indirectly leads to apoptosis in the proximal tubules [17]. Other mechanisms, such as autophagy, necrosis, cell cycle alterations, and others, also contribute to proximal tubular damage [18]. In women, cisplatin-induced ovarian damage results in the loss of healthy ovarian reserve and an increase in atretic follicles [19]. This injury is characterized by a decrease in follicular numbers and reduced estrogen and progesterone activity [20]. In men, cisplatin-treated germ cell cancer is associated with Leydig cell dysfunction, abnormal spermatogenesis, and decreased sperm count. Detectable levels of platinum in the serum can persist for up to 40 months after chemotherapy, further contributing to these reproductive abnormalities [21–25]. When comparing different first-line therapies for advanced urothelial cancer, a systemic review showed that regimens containing cisplatin are associated with higher renal and cardiovascular injury. Other major adverse effects include hematological toxicities [26]. Therefore, cisplatin-induced nephrotoxicity and reproductive damage continue to present significant challenges in clinical practice.

Some studies have demonstrated an increasing interest in agents with nephroprotective or reproductive protective properties [27, 28]. Cimetidine, nilotinib, and magnesium are utilized to decrease cisplatin uptake by the kidneys [29]. Melatonin has the property of ameliorating oxidative stress in the nephron and reproductive system induced by cisplatin [30–33]. However, few studies have investigated the renal and reproductive protective effects of melatonin in mice undergoing chemotherapy.

We hypothesized that the injury caused by cisplatin plus gemcitabine in the nephron and reproductive organs can be reduced if melatonin is administered before and during administration of the chemotherapy cocktail. To test this hypothesis, we examined the effects of melatonin, cisplatin plus gemcitabine, and their combination. We have developed a mouse model with the aim of promoting the potential clinical use of melatonin to mitigate chemotherapy-induced injury. This includes administering melatonin as a pretreatment prior to cisplatin plus gemcitabine administration and then continuing the melatonin treatment for six weeks.

## MATERIALS AND METHODS

### Animal model of chemotherapy-induced organ injury

When considering the doses of melatonin, we referred to past studies. Moradi et al. demonstrated better improvements in recovering injury in a 20 mg/kg melatonin group than in a 10 mg/kg melatonin group, but not in all examined parameters. Following their suggestion for further investigations with higher doses of melatonin, we decided to use 10 mg/kg and 30 mg/kg as our experimental doses [34, 35]. One week of melatonin pretreatment was given before gemcitabine plus cisplatin treatment, as in clinical practice [36]. We then examined the protective effect of melatonin after six weeks of administration, based on previous research [35, 37]. To reflect clinical practice, our experimental design was fixed on using 10 mg/kg and 30 mg/kg of melatonin as our experimental dosage, and we administered the melatonin treatment a week prior to and during the gemcitabine plus cisplatin treatment, which lasted for six weeks.

The animal use protocol was approved by the Chung-Shan Medical University Experimental Animal Center (No. 2554). Male C57BL/6 mice were provided by the National Laboratory Animal Center (NLAC), NARLabs, Taiwan. The mice were housed in a temperature-controlled room with free access to food and water. After one week of habituation, they were randomly distributed into 6 groups, with n = 6 in each group.

**Figure 1:**
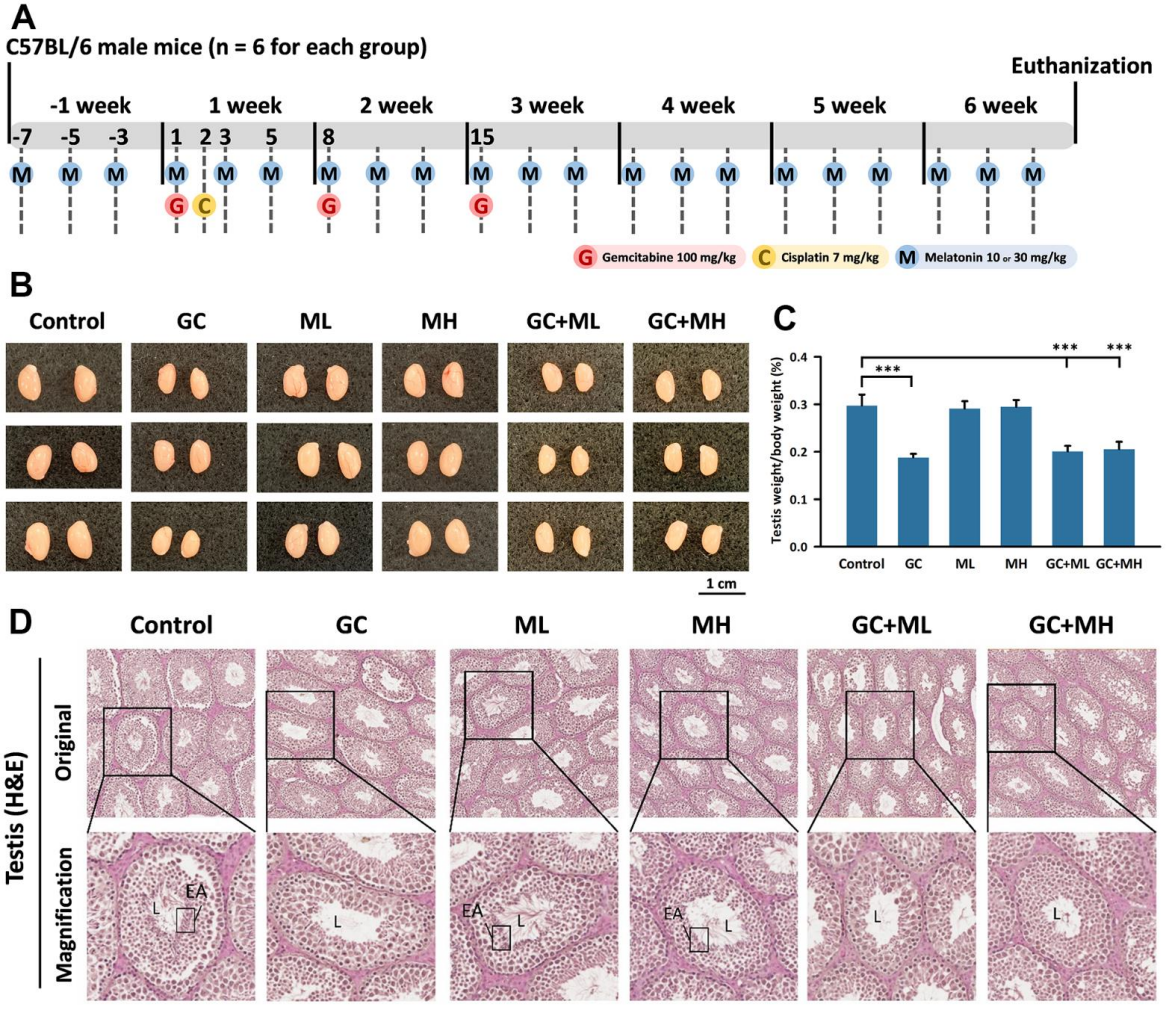
**Effects of melatonin and gemcitabine plus cisplatin on the testis.** The experimental design is illustrated in the timeline (**A**). Representative images of the testes sizes in mice are shown in (**B**). Testis weight relative to body weight under different treatments is presented as a percentage in (**C**, **D**) shows a cross-section of a mouse testicle stained with H&E, where L denotes the lumen, EA represents elongated spermatids, GC refers to Gemcitabine plus Cisplatin, ML represents low-dose melatonin (10 mg/kg), and MH represents high-dose melatonin (30 mg/kg). The data are presented as mean ± SEM, with 6 mice per group. Statistical significance is denoted as *** p < 0.001 compared to the control group.

The entire experiment lasted for 7 weeks, including one week of pretreatment with melatonin and six weeks of treatment sessions (Figure 1A). Mice in the control group received the normal vehicle. The group treated with gemcitabine plus cisplatin (referred to as the GC group) was administered gemcitabine (100 mg/kg) on day 1 of the first three weeks of the treatment session, and cisplatin (7 mg/kg) was given on day 2 of the first week of the treatment session [36]. Mice in the melatonin (ML) group received melatonin (10 mg/kg) on days 1, 3, and 5 during the pretreatment week and throughout the entire treatment session. Mice in the MH (melatonin high-dose) group received a higher dose of melatonin (30 mg/kg) on the same days as the ML group. The GC + ML group and the GC + MH group received combined treatments. Melatonin was purchased from Sigma-Aldrich (Germany), while gemcitabine and cisplatin were purchased from MedChemExpress (USA). All drugs were freshly prepared before administration. At the end of the seventh week, the animals were sacrificed, and their testis, epididymis, and kidneys were weighed and collected for further analysis.

### Histological evaluation of the testis, epididymis, and kidney

The testicle, epididymis, and kidney were fixed in formaldehyde for histological analysis and subsequently embedded in paraffin blocks. The paraffin-embedded tissues were sliced into 3 μm sections, followed by deparaffinization, rehydration, and hematoxylin and eosin staining (Sigma-Aldrich, Germany), as well as trichrome staining (Sigma-Aldrich, Germany). Histological characteristics were examined using TissueFAX Plus (Austria) [38, 39].

### Blood collection and analysis

Mouse blood was collected in centrifuge tubes containing heparin as an anticoagulant. The plasma was subsequently separated and stored at -80° C for further analysis. To assess whether melatonin can mitigate the chemotherapy-induced injury caused by cisplatin and gemcitabine, the levels of reproductive-related hormones and creatinine levels were measured using the standard protocol for each group. Follicle-stimulating hormone (FSH), luteinizing hormone (LH), and testosterone were measured using an ELISA kit (Elabscience, USA), following the manufacturer's instructions.

### Sperm count

Sperm samples were carefully collected from the cauda epididymis of the mice through dissection. In brief, a single cauda epididymis was minced using scissors and transferred to a 1.5 mL tube containing 500 μL of warm medium. After gentle shaking for 5 min, spermatozoa were released from the incision of the cauda epididymis and then incubated in a 37° C, 5% CO2 incubator for 30 min. The supernatant containing sperm was counted three times using a hemocytometer. The sperm count was expressed as 10^5^ cells/mL.

### Statistical analysis

IBM SPSS software (version 20) was utilized for data analysis, and the statistical results were reported as mean ± standard deviation (SD). The Student’s t-test was employed to assess differences between groups, and statistical significance was indicated as follows: * = p < 0.05, ** = p < 0.01, *** = p < 0.001.

### Data availability statement

All data analyzed are included in this article, and additional information is available upon request.

## RESULTS

### Effects of melatonin and gemcitabine plus cisplatin on the testis

To induce a complete treatment cycle, the entire process lasted for 7 weeks. The mice were divided into six groups: a control group, a gemcitabine (100 mg/kg) plus cisplatin (7 mg/kg) group, a low-dose melatonin (10 mg/kg) group, a high-dose melatonin (30 mg/kg) group, a gemcitabine plus cisplatin group with low-dose melatonin, and a gemcitabine plus cisplatin group with high-dose melatonin. Each group consisted of six mice (Figure 1A).

Testicular weight was measured and compared with body weight (Figure 1B, 1C). Compared to the control group (0.297 ± 0.023 %), the testicular weight/body weight significantly decreased in the gemcitabine plus cisplatin group (0.188 ± 0.008 %) (p < 0.001). Low-dose melatonin had minimal adverse effects (0.201 ± 0.012 %) on testicular weight under gemcitabine plus cisplatin treatment, while high-dose melatonin had a more pronounced adverse effect (0.205 ± 0.016%) (Figure 1D).

The testis (H&E) light microscopy results revealed elongated spermatids (EA) in the lumen of the seminiferous tubules. The control group exhibited a robust structure of the basal lamina with round interstitial Leydig cells. Myoid cells lined the basal lamina of the seminiferous tubules, appearing as rod-like shapes. Spermatocytes displayed clear condensed chromatin. Numerous sperm, spermatocyte, and spermatids were present in the seminiferous tubules (Figure 1D).

The lumen of the gemcitabine plus cisplatin group appeared larger and relatively empty due to the absence of elongated spermatids (EA). The basal lamina was thinner compared to the control group, resulting in deformed Leydig cells. The presence of myoid cells was scarce in the gemcitabine plus cisplatin group. Spermatids became smaller, while spermatocytes became larger. The transition in shape from spermatocytes to spermatids became ambiguous, and spermatid numbers significantly diminished, leading to a decrease in elongated spermatids (EA) (Figure 1D).

The testicular pattern was similar in both the low-dose melatonin (ML) and high-dose melatonin (MH) groups. Elongated spermatids could be found in the lumen at a 20× magnification. The basal lamina resembled that of the control group, with round-shaped Leydig cells and rod-shaped myoid cells. Clear chromatin condensation was observed among sperm, spermatocyte, and spermatid cells. Overall, melatonin did not have an effect on the testis in the low-dose and high-dose groups (Figure 1D).

In the gemcitabine plus cisplatin plus low-dose melatonin (GC + ML) group, the seminiferous tubules appeared smaller compared to the control group. The lumen was clear, and elongated spermatids (EA) were barely present. Basal lamina degeneration caused a thinner wall, providing less protection for spermatogonia. The deformity and reduced number of spermatogonia, spermatocytes, and spermatid cells indicated no reversal effect of low-dose melatonin in gemcitabine plus cisplatin treatment (Figure 1D).

The gemcitabine plus cisplatin plus high-dose melatonin (GC + MH) group indicated a slight protective effect of melatonin. More spermatogonia, spermatocytes, and spermatid cells were present in the seminiferous tubules. Slightly greater numbers of elongated spermatids (EA) were present in the lumen compared to the GC + ML group. Leydig cells were fewer than in the control group, but more abundant than in the GC or GC + ML group. The deformity caused by gemcitabine and cisplatin was still noticeable, but the spermatids were greater in number than those in the GC or GC + ML groups. High-dose melatonin demonstrated a slightly protective effect on the testis (Figure 1D).

### Effects of melatonin and gemcitabine plus cisplatin on the epididymis

We referred to the “Diagnosis and Treatment of Infertility in Men: AUA/ASRM Guideline (2020)” as a primary resource [40]. In clinical practice, physicians conduct a comprehensive physical examination and assess the patient’s reproductive history, while also performing a semen analysis, with a gap of at least one month between. Furthermore, hormone tests, including FSH, LH, and testosterone (TT) levels, are conducted. In cases where TT levels are low, repeating the testosterone examination is recommended. Further assessments of free testosterone, bioavailable testosterone, LH, and prolactin levels are also recommended [41]. FSH stimulates spermatogenesis, whereas LH promotes testosterone synthesis. A low sperm concentration indicates a higher likelihood of infertility, while low FSH and testosterone levels adversely affect spermatogenesis.

**Figure 2:**
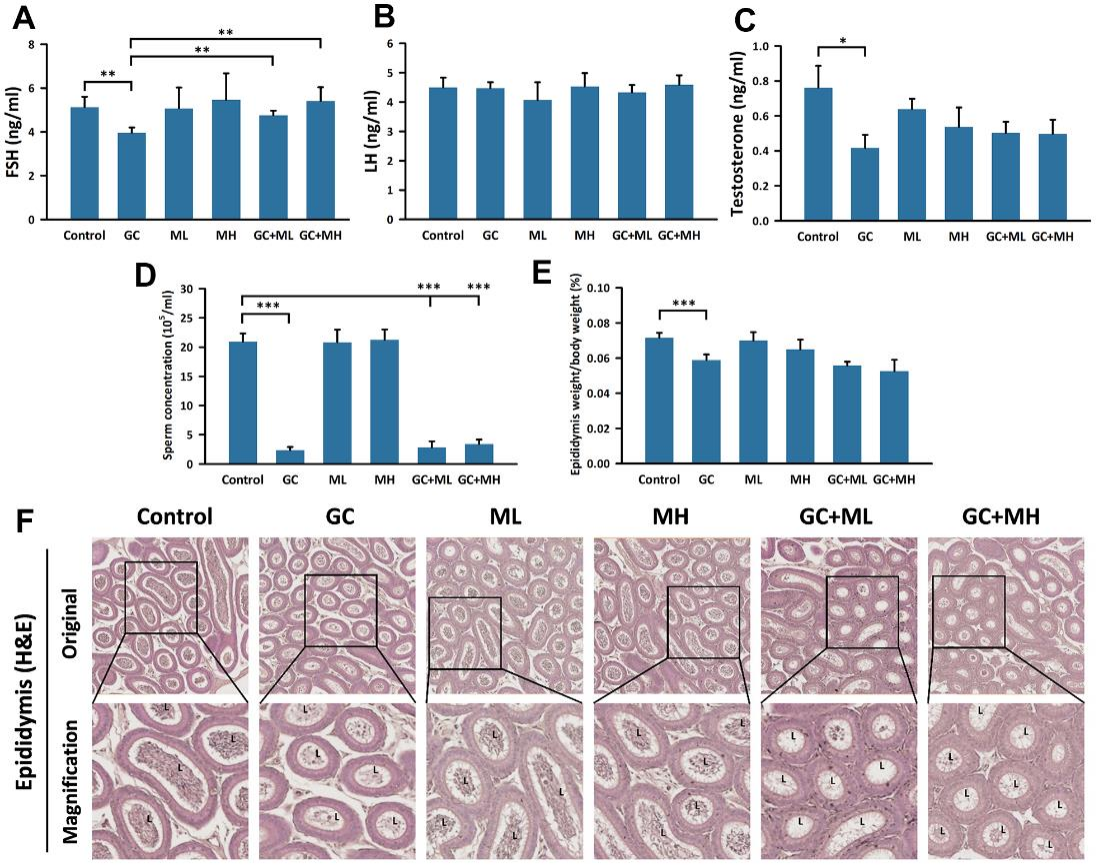
**Effects of melatonin and gemcitabine plus cisplatin on the epididymis.** The histogram presents the percentage of (**A**) FSH detection in serum, (**B**) LH detection in serum, (**C**) testosterone detection in serum, (**D**) sperm concentration in the epididymis, and (**E**) epididymis weight relative to body weight under different treatments. Additionally, (**F**) a cross-section of the mouse epididymis stained with H&E is shown. GC refers to gemcitabine plus cisplatin, ML represents low dose (10 mg/kg), and MH indicates high-dose (30 mg/kg) melatonin. The data are presented as mean ± SEM, with 6 mice per group (N = 6 mice per group). Statistical significance is denoted as * p < 0.05, ** p < 0.01, and *** p < 0.001 compared to the control or GC group.

Serum FSH, serum LH, serum total TT, and sperm concentrations were analyzed (Figure 2A–2D). The serum FSH level was lower in the GC group (3.96 ± 0.24 ng/ml) than in the control group (5.13 ± 0.48 ng/ml) (p < 0.005). By contrast, the FSH levels were significantly higher in the GC plus ML group (4.76 ± 0.21 ng/ml) than in the GC group (p < 0.005). The FSH levels were also significantly higher in the GC plus MH group (5.41 ± 0.63 ng/ml) than in the GC group. No significant changes were detected in the serum LH levels among the groups. Both the ML group (4.07 ± 0.6 ng/ml) and the GC plus ML group (4.33 ± 0.26 ng/ml) showed a slight decrease in serum LH levels compared to the control group (4.5 ± 0.34 ng/ml). However, the serum TT level decreased in the GC group (0.42 ± 0.08 ng/ml). The addition of melatonin had a slightly adverse effect on GC treatment. The serum TT level was slightly higher in the GC plus ML group (0.5 ± 0.06 ng/ml) than in the GC group (0.42 ± 0.08 ng/ml), which was consistent with the TT level in the GC plus MH group (0.5 ± 0.08 ng/ml). Regarding sperm concentration, the GC treatment significantly reduced sperm concentration (2.35 × 10^5^/ml) compared to the control group (20.93 × 10^5^/ml). Melatonin slightly reversed the GC treatment effect, and the GC plus MH group exhibited a slightly higher sperm concentration (3.4 × 10^5^/ml).

The H&E staining results are presented in Figure 2F, and the weight of the epididymis/body weight is shown as a percentage in Figure 2E. The weight of the epididymis was lower in the GC group (0.059 ± 0.003%) than in the control group (0.072 ± 0.003%) (p < 0.001). However, a high dose of melatonin had no protective effect on the weight of the injured epididymis (0.053 ± 0.006%). The lumen of the epididymis was smaller in all the GC groups than in the non-GC group. The control, ML, and NH groups contained numerous spermatozoa in the lumen, whereas few spermatozoa were found in the GC, GC plus ML, and GC plus MH groups.

### Effects of melatonin and gemcitabine plus cisplatin on the kidney

**Figure 3:**
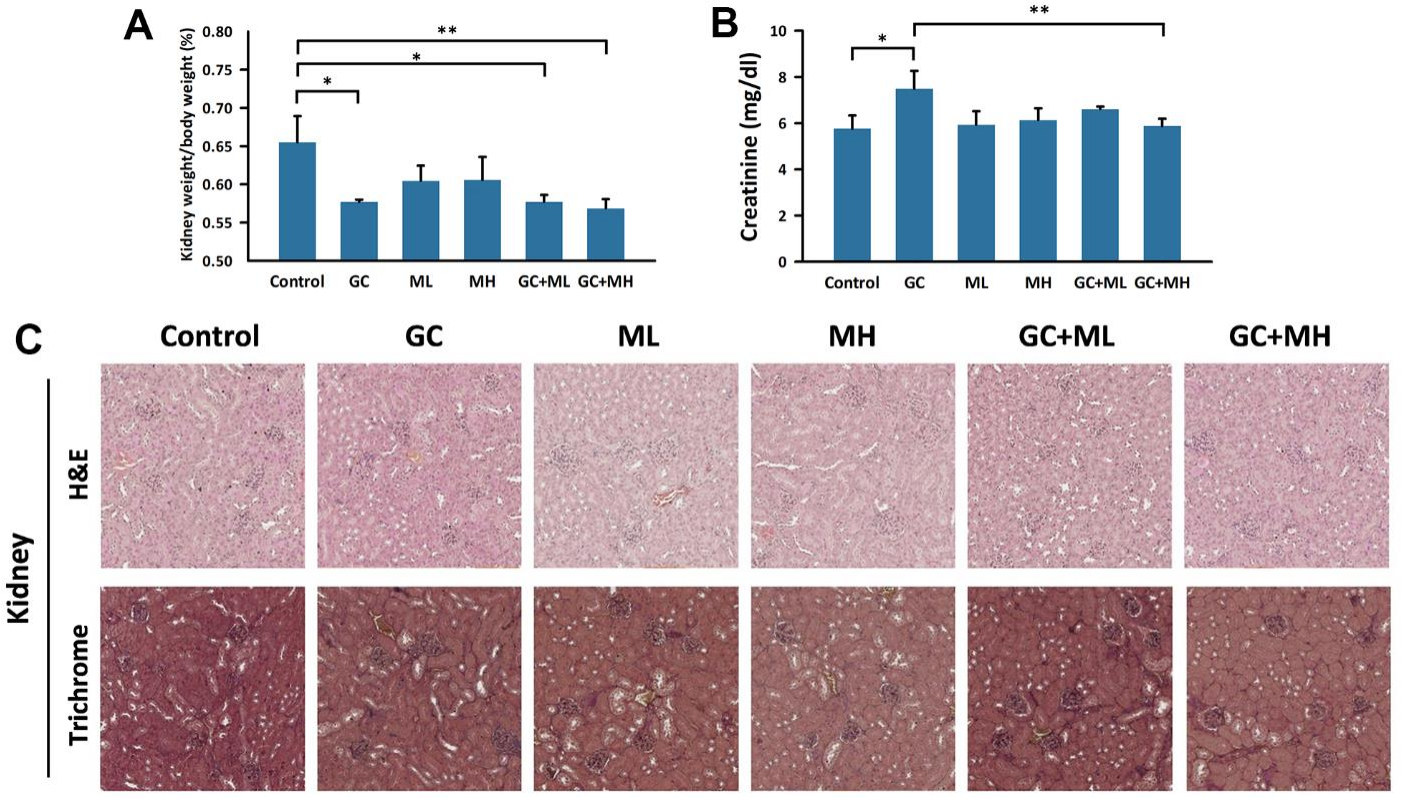
**Effects of melatonin and gemcitabine plus cisplatin on the kidney.** The histogram illustrates the percentage of (**A**) kidney weight/body weight under different treatments and (**B**) detection of creatinine in the serum. A tangential section of the mouse kidney stained with H&E and trichrome is shown in (**C**). GC refers to gemcitabine plus cisplatin, ML represents low dose (10 mg/kg) melatonin, and MH represents high dose (30 mg/kg) melatonin. The data are presented as mean ± SEM, with 6 mice per group (N = 6 mice per group). * p < 0.05, ** p < 0.01 compared to the control or GC group.

The histological section of the kidney is shown in Figure 3C. The renal cortex contains a renal corpuscle surrounded by the visceral and parietal epithelium of Bowman’s capsule, while the tubules extend from the medulla. H&E staining did not reveal any observable structural changes histologically. However, Masson’s trichrome stain revealed increased fibrosis in Bowman’s capsule in the groups receiving gemcitabine plus cisplatin. Low-dose melatonin did not show a protective effect, while high-dose melatonin showed a slight protective effect.

The kidney weight was significantly lower in the GC group (0.577 ± 0.003%) than in the control group (0.655 ± 0.034%) (p = 0.019). However, the administration of either low- or high-dose melatonin in combination with GC did not demonstrate a dose-dependent effect on the recovery of kidney weight (GC+ML: 0.577 ± 0.009%; GC+MH: 0.569 ± 0.012%). Serum creatinine levels were significantly higher in the GC group (7.49 ± 0.77 mg/dL) than in the control group (5.76 ± 0.57 mg/dL) but were significantly lower in the GC + MH group (5.87 ± 0.32 mg/dL), and approached the level in the control group (5.76 ± 0.57 mg/dL).

## DISCUSSION

In our study, we evaluated the reproductive injury caused by the administration of gemcitabine and cisplatin at the end of the sixth week. We observed significant decreases in testis weight, epididymis weight, and sperm concentration after the administration of gemcitabine plus cisplatin to the GC group. H&E staining of tissues from the GC group showed changes in the germinal epithelium, such as a thinner basal lamina and deformed Leydig cells. The serum levels of reproductive-related hormones, including FSH and testosterone, were reduced in the GC group, while the serum level of LH remained unchanged compared to the control group. Numerous *in vivo* studies have consistently reported cisplatin-induced injuries to the testis and epididymis [30, 42, 43]. The measurements by Eren et al. of the levels of glutathione (GSH), superoxide dismutase (SOD), and malondialdehyde (MDA) also demonstrated that cisplatin can induce testicular injury through the oxidative stress pathway [44]. Our observations regarding changes in reproductive-related hormones were similar to those of a previous study, but they may be influenced by different cisplatin doses and evaluation periods [45].

We discovered that administration of high-dose melatonin (30 mg/kg) has a slightly adverse effect on testicular tissue treated with GC, leading to an increase in elongated spermatids and spermatids. In comparison to the GC group, melatonin significantly reverses the effects of GC on serum FSH levels and slightly restores the serum testosterone level. However, melatonin has no demonstrable effects on serum LH, sperm concentration, or the epididymis. Previous studies have demonstrated an ameliorating effect of melatonin on oxidative stress in injured testes, as observed by maintenance of the blood–testis barrier (BTB), improving sperm quality, and reducing capillary permeability [30, 46, 47].

The GC group showed decreased kidney weight and increased serum creatinine levels, indicating that cisplatin causes kidney injury. The greater accumulation of cisplatin in tubular cells is the primary reason underlying the observed nephrotoxicity [1]. Cisplatin enters renal cells through Ctrl and OCT transporters, subsequently activating its toxicity within those cells [29]. Thus, the mechanism of cisplatin toxicity mainly involves necrosis and apoptosis [29]. The caspase-dependent apoptotic pathway involves the activation of Bax and Bak, mitochondrial dysfunction, and cytochrome c release. Additionally, caspase-independent apoptotic pathways, such as AIF, Smac, and PUMA-a, contribute to cisplatin-induced toxicity. Other pathways involved in toxicity include the ER pathway, regulation of cyclin-dependent kinases (CDKs), the MAPK pathway, oxidative stress, and inflammation [48, 49]. Cisplatin induces oxidative stress by disrupting glutathione levels, mitochondrial function, or the cytoprotective response [50]. Yu et al. described shifts in glycolysis, the pentose phosphate pathway, and the citric acid cycle that occur *in vitro* due to cisplatin-induced oxidative stress [51].

Here, we observed elevated serum creatinine levels in the GC group, but this elevation was reversed by melatonin administration. Melatonin has been reported to inhibit NF-κB/P65 translocation, thereby impeding the inflammatory process [52, 53]. As previously mentioned, oxidative stress plays a role in apoptotic-induced mechanisms. Melatonin promotes the production of glutathione peroxidase (GPx), which is associated with the non-free radical metabolism of H_2_O_2_ [54, 55]_._ Additionally, melatonin reduces the activity of nitric oxide synthetase (NOS), which converts L-arginine into L-citrulline and NO [56]. Another unique biochemical characteristic of melatonin is its direct enhancement of mitochondrial electron transport chain (ETC) activity through non-receptor-mediated scavenging abilities [57]. Sun et al. demonstrated that melatonin counteracts the decrease in PPARα induced by cisplatin in renal tubular epithelial cells [32]. Moreover, Kim et al. showed that melatonin inhibits apoptosis and necroptosis in cisplatin-induced acute kidney injury [58]. Previous research on nephrotoxicity and reproductive agents has identified melatonin as a relatively safe treatment agent [59]. We utilized clinical chemotherapy as a reference to develop an animal model for incorporation of the administration of gemcitabine plus cisplatin, melatonin pretreatment, and melatonin treatment at doses of 10 mg/kg and 30 mg/kg. We also referred to prior investigations in our animal model. We used the same administration period for GC on days one and two in the first two weeks, as described previously for the animal model used by Miyake et al. [60]. Nevertheless, our study has certain limitations. The first was the use of a fixed time schedule for the different groups, as this prevented us from capturing time-related changes and from detecting whether a longer intervention might change the outcome. The gemcitabine and cisplatin dosages remained fixed throughout the study, and we had limited observation data. Previous studies have included markers associated with oxidative stress, proteomics evaluations, and comprehensive analysis of reproductive epithelia [30, 44]. Another limitation lies in the fact that the C57BL/6 male mouse model may not accurately represent a female mouse model or other animal models. Another important point to note is that the animal model itself cannot fully replicate human physiology, and the findings, especially the long-term effects of melatonin, may not fully translate to clinical outcomes in humans.

## CONCLUSIONS

We have demonstrated a potential protective effect of melatonin, albeit slight, against cisplatin-induced reproductive and renal injury. Our findings using the C57BL/6 male mouse model indicate that melatonin has opposing effects on testicular weight, testicular histological injury, serum reproductive hormones, and serum creatinine to those induced by GC treatment. Therefore, our investigation showed a partial protective effect of melatonin in restoring reproductive function and regulating creatinine levels, but further study is needed to conclusively establish a protective effect or a partially protective effect of melatonin against cisplatin-induced injury *in vivo*.
